# Persistence of back pain symptoms after pregnancy and bone mineral density changes as measured by quantitative ultrasound - a two year longitudinal follow up study

**DOI:** 10.1186/1471-2474-12-55

**Published:** 2011-02-28

**Authors:** William WK To, Margaret WN Wong

**Affiliations:** 1 Department of Obstetrics & Gynaecology, United Christian Hospital, Hip Wo Street, Kwun Tong, Hong Kong, PR China; 2 Department of Orthopaedics & Traumatology, Prince of Wales Hospital, Shatin, Hong Kong, PR China

## Abstract

**Background:**

Previous research has shown a loss of bone mineral density (BMD) during pregnancy. This loss has been correlated to the occurrence of back pain symptoms during pregnancy. The objective of this study was to evaluate whether persistence of back pain symptoms 2 years after pregnancy could be associated with BMD changes as measured by quantitative USG of the os calcis.

**Methods:**

A cohort of patients who reported significant back pain symptoms during pregnancy were surveyed for persistent back pain symptoms 24 to 28 months after the index pregnancy. Os calcis BMD was measured by quantitative ultrasound and compared with the BMD values during pregnancy.

**Results:**

A cohort of 60 women who had reported significant back pain symptoms in their index pregnancy completed a 24-28 months follow-up survey and BMD reassessment. Persistence of significant back pain symptoms was seen in 24 (40%) of this cohort. These women had higher BMD loss during pregnancy compared to those without further pain (0.047 Vs 0.030 g/cm^2^; p = 0.03). Those that remained pain free after pregnancy appeared to have completely recovered their BMD loss in pregnancy, while those with persistent pain had lower BMD values (ΔBMD - 0.007 Vs - 0.025 g/cm^2^; p = 0.023) compared to their early pregnancy values.

**Conclusion:**

Persistence of back pain symptoms after pregnancy could be related to an inability to recover fully from BMD loss during the index pregnancy.

## Background

Back pain is a common complaint in pregnancy. The incidence of significant back pain during pregnancy varies widely in different populations from 20-60% [1-3]. While the causative factors of back pain during pregnancy are likely to be multi-factorial and heterogeneous, a positive association between bone loss and pregnancy related back and pelvic pain symptoms has been proposed. Decreased femoral bone density was associated with hip pain in the immediate postpartum period [4], while a greater fall in os calcis BMD has been shown to be correlated to back pain symptoms during pregnancy [5]. The incidence of persistent back pain symptoms after pregnancy varied from the disappearance of pain in over 60% within 2 days after delivery [6], to as high as 82% having persistent pain at 18 months in those with recurrent back pain from previous pregnancies [7]. An overall incidence of around 21% at 2 years after delivery has been reported [3]. Various risk factors have been ascribed to the persistence of pain after the index pregnancy, including history of back pain [8] or other epidemiological factors such as smoking or occupation [9], but the role of postpartum BMD loss or osteoporosis [10] still remains controversial.

This study aims at observing the postpartum BMD changes in a longitudinal cohort of pregnant women who had reported significant back pain during pregnancy and correlating such changes with the incidence of persistent back pain symptoms 24 months or more after pregnancy using quantitative USG at the os calcis. The findings would help to evaluate whether the extent of recovery of the BMD loss that occurred during pregnancy would be protective against persistent back pain symptoms.

## Methods

### Pregnancy Cohort

In the index pregnancy cohort, consecutive patients booked at a general obstetric clinic were prospectively recruited for the study over a twelve-month period. Routine antenatal care was offered in accordance with our service protocol. Written consent was obtained at the time of recruitment, and basic epidemiological data, including early pregnancy weight and height were recorded. Quantitative ultrasound bone density measurements were performed at the os calcis bilaterally at booking between 16 to 20 weeks, and in the third trimester between 36-38 weeks. All measurements were done using the Sahara Clinical Bone Sonometer system (Hologic), a waterless portable system that involved direct contact of the probe with the heel through elastomer pads and a specific ultrasound coupling gel. The patient was positioned as recommended by the manufacturer, seated in an upright position in a stable chair without wheels. The patient foot was stabilized using a specific foot guard to ensure that the focus between the ultrasound probes corresponded to the region of interest at the os calcis. All patients were allowed 30 minutes in the clinic before measurement to allow ambient skin temperature to be attained at the heel. Measurements were made bilaterally. The system measures BUA, SOS and then used these parameters to generate a simulated BMD. This computer calculated BMD was used in the calculations. The coefficient of variation of the system was quoted as 2-3% by the manufacturer and was in accordance with the data of the investigators using the system in previous similar studies. In addition to weight, body fat percentage assay was also performed in each of these occasions using a Tanita 500 bio-impedance system. Patients who had known medical conditions or who were on long-term medications known to affect bone density values, such as steroids or thyroid drugs, and those who delivered preterm before 36 weeks, were excluded from the final analysis. In addition, patients who have known chronic back pain that required regular medical follow-up or treatment, known spinal deformities and previous surgical intervention for back pain were also excluded. The patients were then surveyed for back pain symptoms during the index pregnancy by means of a structured self-administered questionnaire in the early postpartum period within the first 3 days before their discharge from hospital. Women who reported positive pain symptoms at any stage in her pregnancy were requested to fill in a pain distribution chart from recall. A visual analog scale of pain intensity was also provided to classify mild, moderate or severe back pain symptoms. Patients who reported at least moderate pain for more than 3 consecutive days within the pregnancy, or who required additional medical consultation, sick leave or formal treatment because of back pain during their pregnancy were considered as positive. Those who complained of only mild pain of transient durations were considered negative for back pain symptoms. The correlation of presence/absence of back pain in pregnancy and the interval BMD changes in pregnancy has previously been published [5].

### Two Year Reassessment Cohort

All patients were resurveyed for back pain symptoms between 24-28 months after delivery using a mailed questionnaire, with a format similar to the early postnatal version. Those who reported at least moderate pain for more than 3 consecutive days within the past 6 months of the questionnaire, or who required medical consultation, sick leave or treatment because of back pain during the past 6 months were considered as positive. Those with only mild or transient symptoms that did not require medical consultation or treatment were regarded as negative. Those who already had further pregnancies at the time of the survey were excluded from the analysis.

Patients who responded to the 24-28 month survey were invited to attend a special clinic session for repeat BMD measurements to compare with their pregnancy values. At this 2-year post delivery evaluation, the patients were given a standard interview to record their menstrual status, last menstrual dates and breast feeding status. Any other remarkable medical conditions or the need for long term medications were also noted. Care was taken to exclude the possibility of pregnancy in these subjects, and if confirmed, these were excluded from further investigations and analysis. Anthropometric and quantitative ultrasound measurements were performed using an identical protocol as the assessments during pregnancy.

The current study cohort consisted of women who had reported positive back pain symptoms in their index pregnancy, and who completed the 2-year post delivery questionnaire and BMD assessment. Interval changes in body weight, body fat percentage, and os calcis BMD were calculated and correlated with the presence and absence of persistent back pain, as well as with previous pregnancy changes. A regression model was established to evaluate the inter-correlation of these parameters and persistent back pain symptoms. A p-value of <0.05 was considered significant. Data was analyzed using the SPSS version 13.0 (SPSS, Chicago, IL, USA). The study was approved of by the Ethics Committee of the cluster hospital board.

## Results

**Figure 1:**
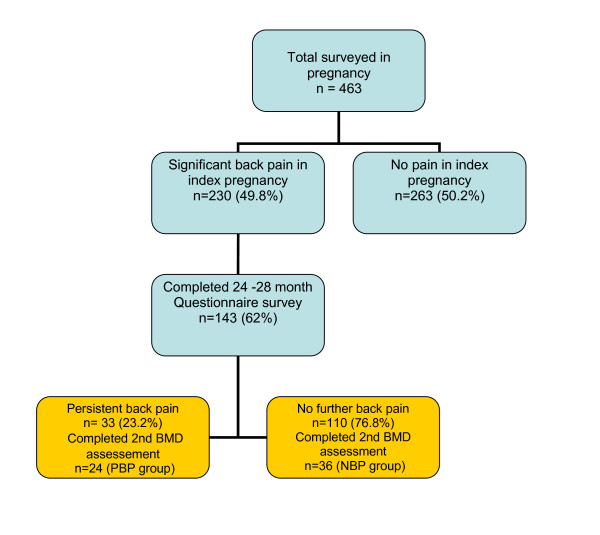
**Flow chart of women with or without persistent back pain included in the two-year longitudinal study**. PBK, persistent back pain; NBP, no persistent back pain.

Of 463 patients recruited in pregnancy in the original cohort, 230 (49.8%) reported one or more episodes of significant back pain during pregnancy. Of these, 143 (62%) with no further pregnancies completed the 24-28 months questionnaire follow-up survey, and persistence of significant back pain symptoms was seen in 33 (23.2%). Of those that completed the 2-year survey, 60 (41.9%) were available to complete the follow-up BMD assessment, which included 24 categorized as positive for persistent back pain, and 36 without further back pain symptoms (Figure 1). This final cohort of 60 women was used for further analysis in the current study.

The mean loss in BMD from early to late gestation in the index pregnancy of this cohort was 0.373 g/cm^2 ^(SD 0.029), representing around 5% of the early pregnancy value. A marginally loss of BMD was seen when the 2-year post delivery measurement was compared with the early pregnancy value (- 0.022 g/cm^2^, SD 0.04). On the other hand, body weight, body fat percentage and body mass index significantly increased from early to late pregnancy, but fell again at the 2-year post delivery survey. A positive gain was seen in all these parameters when the 2-year post delivery measurement was compared with the early pregnancy value (Table 1).

**Table 1:** Changes in basic anthropometric parameters during pregnancy and 24-28 months after delivery (n = 60)

	Early Pregnancy (< 20 weeks) (SD)	Late Third Trimester (36-38 weeks) (SD)	Two years post delivery (SD)	P-value by ANOVA
Weight (kg)	56 (7.38)	65.5 (7.67)	59.3 (7.43)	< 0.001
Body Mass Index (kg/cm^2^)	22.7 (2.84)	26.6 (2.79)	24.1 (2.91)	< 0.001
Body Fat Composition (%)	29.4 (4.75)	37.3 (4.52)	32 (4.93)	< 0.001
Mean os calcis BMD (g/cm^2^)	0.638 (0.13)	0.601 (0.116)	0.616 (0.123)	< 0.001

SD = standard deviation

The final cohort was then divided into the group with significant persistent back pain (PBP group, n = 24)) and the group without further back pain (NBP group, n = 36) at the 24-28 months assessment. The PBP group had higher early pregnancy BMD (0.686 Vs 0.605 g/cm^2^, p = 0.02) but also had higher BMD loss during pregnancy (0.047 Vs 0.030 g/cm^2^, p = 0.03) when compared to the NBP group. The PBK group also had higher weight gain at 2 years (3.68 Vs 2.86 kg, p = 0.031), and a higher net loss of BMD (-0.025 Vs -0.007 g/cm^2^, p = 0.023) at the 24-28 month assessment when compared to early pregnancy values. There was no difference in the duration of lactation in the index pregnancy between the two groups (Table 2). The NBP group appeared to have almost completely recovered their BMD loss in pregnancy, the BMD values at 2 years after delivery was nearly identical to the early pregnancy values (Table 2).

**Table 2:** Comparison of anthropometric and BMD differences in those with or without persistent pain at 24-28 months

	PBP group (n = 24)	NBP group (n = 36)	p-value; MD (95% CI)
Age (years)	33.3 (3.14)	32.2 (2.82)	0.22; 0.95 (-0.59 to 2.51)
Height (cm)	157 (5.2)	156 (6.2)	0.67; 0.66 (-2.43 to 3.75)
Early pregnancy weight (kg)	54.5 (6.83)	57 (7.66)	0.21; -2.44 (-6.32 to 1.42)
Early pregnancy BMI (kg/m^2^)	22 (2.55)	23.2 (2.96)	0.11; -1.19 (-2.67 to 0.28)
Early pregnancy body fat percentage	28.7 (4.27)	29.8 (5.01)	0.66; -0.30 (-3.53 to 1.5)
Early pregnancy BMD (g/cm^2^)	0.686 (0.135)	0.605 (0.124)	0.021; - 0.081 (0.001 to 0.12)
Pregnancy weight gain (kg)	9.55 (2.35)	9.43 (3.37)	0.88; 0.11 (-1.47 to 1.70)
Pregnancy body fat accumulation (%)	7.75 (1.89)	8.05 (3.02)	0.66; -0.30 (-1.69 to 1.08)
Pregnancy BMD loss (g/cm^2^)	0.0472(0.018)	0.0306(0.033)	0.034; 0.0165 (-0.031 to -0.013)
Lactation duration in index pregnancy (weeks)	8.8 (4.5)	8.1 (3.6)	0.50; 0.72 (-1.4 to 2.85)
Weight change at 2 yrs post delivery (kg)	3.68 (1.84)	2.86 (1.02)	0.031; 0.82 (0.076 to 1.57)
Body fat change 2 yrs post delivery (%)	2.33 (1.34)	2.68 (1.85)	0.42; -0.35 (-1.23 to 0.53)
BMD change 2 yrs post delivery (g/cm^2^)	-0.0257 (0.029)	-0.0007 (0.028)	0.023; -0.017 (-0.033 to-0.0025)

MD = mean difference

CI = confidence interval

The correlation between the early BMD values and the 24-28 month post delivery values were highly significant and reliable (Pearson correlation coefficient 0.95, p < 0.001 with 2-tailed analysis), as was the correlation between the late pregnancy BMD values and the 24-28 month post delivery value (Pearson correlation coefficient 0.97, p < 0.001 with 2 -tailed analysis).

A logistic regression model was constructed using the presence or absence of persistent back pain symptoms at the 24-28 months assessment as the dependent variable against all likely confounding continuous variables. Of the 4 variables found to be significant on univariate analysis, only weight gain at 2 years post delivery (p = 0.03) and BMD changes at 2 years post delivery (p = 0.03) remained in the equation, while the effects of early pregnancy BMD values and pregnancy BMD loss disappeared (Table 3). In qualitative terms, more weight gain at 2 years after delivery are associated with persistent back pain, while a net positive balance in BMD at 2 years was protective against persistent back pain symptoms.

**Table 3:** Stepwise logistic regression using persistence of significant post delivery back pain as dependent variable

Variable	B	**S.E**.	Wald	Significance	Odds ratio	95% CI
***Significant variables***						
Weight Gain at 2 years post delivery	-0.653	0.303	4.62	0.03	1.92	1.05 to 3.48
BMD change at 2 years post delivery	-21.9	10.26	4.55	0.03	0.11	0.01 to 0.96
						
***Excluded variables***						
Age	0.0529	0.113	0.203	0.65	1.05	0.84 to1.32
Early pregnancy BMI	-0.406	0.238	2.90	0.08	0.66	0.41 to 1.06
Early pregnancy fat percentage	0.131	0.125	1.09	0.29	1.14	0.89 to 1.45
Early pregnancy BMD	0.208	3.944	0.002	0.95	1.23	0.03 to 5.7
Weight gain in pregnancy	-0.031	0.129	0.058	0.80	0.96	0.75 to 1.24
Fat gain in pregnancy	-0.253	0.211	1.434	0.23	0.77	0.51 to 1.17
Pregnancy BMD loss	25.26	16.97	2.21	0.13	0.37	0.06 to 8.91
Fat change at 2 years post delivery	-0.406	0.267	2.32	0.13	0.67	0.39 to 1.13

CI = confidence interval

## Discussion

The data presented in this study confirmed a demonstrable progressive fall in BMD at the os calcis as measured by quantitative ultrasound from early to late pregnancy. The mean decrease in BMD was around 5.5% of the early pregnancy BMD value, and was consistent with previous studies utilizing various means to measure BMD loss in pregnancy [11,12], including quantitative ultrasound measurements using different [13,14] or similar systems [5,15,16]. The incidence of back pain symptoms of around 50% that was found in the current study was in line with what was reported in the literature [1-3]. The incidence of persistent back pain symptoms of around 20% at 2 years was also compatible with the data in the literature [3,17-19]. In addition, the current data was able to support an association between BMD loss during pregnancy, the degree of recovery of BMD loss after pregnancy and the persistence of back pain symptoms after the delivery.

Previous studies evaluating the risk factors for persistence of back pain in pregnancy focused on history of back pain [3,18], older age [3], younger age and higher weight [8], maternal smoking [9], the pattern of pain during the index pregnancy [20], as well as other psychosocial factors [8], but the role of postpartum BMD changes to persistence of back pain symptoms have yet to be studied in detail. Pregnancy has been documented to be a state of marked enhancement of bone turnover [21], during which a significant loss in BMD could be clearly demonstrated by direct methods, including standard dual-energy X-ray absorptiometry (DXA) [11,22] and quantitative ultrasound [13-16]. This BMD loss is thought largely to be reversible in the long run [23,24]. While the role of BMD changes in relation to back pain symptoms during pregnancy have been explored [5,10], the longer term effects of such BMD loss in relation to persistence of back pain remains controversial. Previous studies have observed that a significant proportion of women who had documented back pain symptoms in pregnancy will be predisposed to have continued symptoms in subsequent years [18,19]. Our findings of higher BMD loss during pregnancy and inability to attain complete recovery of this loss after 2 years in the group with persistent back pain would suggest a relationship between persistent BMD loss and persistent back pain. It would be of interest to evaluate whether the group of women who would continue to have severe back pain symptoms in later life would be more prone to develop clinical osteoporosis than those without.

The possible mechanisms relating BMD loss and back pain remains elusive, as even in women with severe persistent back pain, the symptoms were only rarely associated with vertebral fractures or demonstrable radiological abnormalities [25]. Others would ascribe the back pain to biomechanical factors rather than to BMD loss [20], and that the BMD loss could theoretically be the result of immobilization or reduction in exercise levels because of persistent pain symptoms. However, quantitative BMD loss short of demonstrable fractures has also been associated with back, pelvic pain, as well as with hip pain symptoms [5,10]. Lower BMD values during pregnancy have been associated with a higher incidence of back and pelvic pain symptoms [14]. An association between decreased femoral bone density or transient osteoporosis of the hip and hip pain during pregnancy and in the immediate postpartum period has been reported [26-28]. Thus, mild forms of pregnancy osteoporosis might pass undiagnosed clinically, but could be associated with pain symptoms. In addition, in this cohort, we have not studied calcium intake or vitamin D status and the impact of these parameters on BMD loss during or after pregnancy. Further studies to address these issues would help to explain the pathophysiology underlying BMD recovery after pregnancy and delivery and the relationship to back pain.

In this cohort, we have observed that the persistent back pain group had higher early pregnancy BMD, but also higher BMD loss during pregnancy, as compared to those with no persistent pain. In our previous study [5], we have found that those with higher BMD loss in pregnancy actually tend to have higher BMD to start off with in early pregnancy, while those with borderline low BMD in early pregnancy apparently preserved BMD better and thus have lower BMD loss in pregnancy. As those with persistent back pain after pregnancy were also more likely to have higher BMD loss during pregnancy, epidemiologically, this could lead to the observation that those with persistent back pain having significantly higher early pregnancy BMD.

There were certain limitations to this study. While we have been able to survey the incidence of persistent back pain symptoms in around 55% of the original cohort, we were able to obtain BMD findings in only around 26% of our original cohort that reported back pain in pregnancy (60/230). It could be seen that only around 33% of those without further pain were available for the follow-up BMD assessment, while up to 73% of those with further pain underwent the BMD assessment. However, secondary analysis of indicative parameters such as basic epidemiological characteristics, BMD loss in pregnancy and the incidence of persistent back pain between the group that completed the follow-up study and those that defaulted did not show any significant differences. We thus believe that the data of the group presented here should be representative of the entire cohort. In addition, while the relatively small sample size in the final cohort could be underpowered to show differences in secondary parameters such as body fat changes at two years follow-up, the current cohort was already able to show consistent and significant differences in primary outcome parameters such as BMD loss during pregnancy and at 2 years post delivery between the two groups.

Quantitative ultrasound measurements of BMD have in general demonstrated good correlation with DXA measurements and are comparable to DXA in the prediction of clinical osteoporosis and fractures. Serial longitudinal comparisons could be affected by a relatively large coefficient of variation of 2-3% inherent in these quantitative ultrasound systems, particularly when the absolute difference to be measured is of magnitudes smaller than the coefficient of variation. However, as the magnitude of measurable BMD loss during pregnancy was substantial larger (5-7%) than the projected least significant change (LSC) that could be measured with the method, we believe that such measurements should be valid. Previous studies and our own data have demonstrated that the results of such quantitative ultrasound systems appear to be consistent and reproducible. [13-16]. In addition, when comparing the pregnancy BMD values and the 2-year follow-up values, we were able to find very high correlation coefficients despite the long time intervals between measurements This should be good evidence to support the reproducibility of such BMD measurements over time for any single individual. On the other hand, it could also be argued that after pregnancy, other measurement methods such as standard DXA for the axial skeleton or peripheral quantitative computerized tomography for the appendicular skeleton, which should have lower coefficients of variation, could provide more precise data. However, due to the theoretical risks of radiological exposure during pregnancy, such methods could only be used after delivery and direct correlation with data on BMD changes of the same skeletal site during pregnancy would not be possible. We have thus chosen to use the same method of measurement after pregnancy in order to compare directly with pregnancy values despite the limitations of such measurement methodology. Our data have shown that quantitative ultrasound is a viable method for monitoring the recovery of BMD after delivery to its pre- or early pregnancy states.

## Conclusions

In summary, the findings in this study supported a correlation of BMD loss as well as the degree of recovery of this loss as measured by quantitative ultrasound and persistent back pain symptoms in pregnancy. Future larger scale studies involving serial measurements of BMD at different skeletal sites using methods to correlate with persistent back pain symptoms should be warranted. The long term implications of the ability to recover the BMD loss in pregnancy in terms of menopausal bone health and risks of osteoporosis would also need to be explored.

## Competing interests

The authors declare that they have no competing interests.

## Authors' contributions

Both authors conceived of the study, participated in the design and coordination of the study. Both authors participated in the data analysis and preparation of the manuscript. Both authors read and approved the final manuscript

## Pre-publication history

The pre-publication history for this paper can be accessed here:

http://www.biomedcentral.com/1471-2474/12/55/prepub
